# Tranexamic acid for the prevention and treatment of bleeding in surgery, trauma and bleeding disorders: a narrative review

**DOI:** 10.1186/s12959-021-00303-9

**Published:** 2021-08-11

**Authors:** Anna Ockerman, Thomas Vanassche, Melisa Garip, Christophe Vandenbriele, Matthias M Engelen, Jeroen Martens, Constantinus Politis, Reinhilde Jacobs, Peter Verhamme

**Affiliations:** 1 Department of Imaging and Pathology, KU Leuven, OMFS-IMPATH Research Group, Leuven, Belgium; 2 Department of Oral & Maxillofacial Surgery, University Hospitals Leuven, Leuven, Belgium; 3 Department of Cardiovascular Sciences, KU Leuven, Leuven, Belgium; 4 Department of Dental Medicine, Karolinska Institutet, Stockholm, Sweden

## Abstract

**Objectives:**

We review the evidence for tranexamic acid (TXA) for the treatment and prevention of bleeding caused by surgery, trauma and bleeding disorders. We highlight therapeutic areas where evidence is lacking and discuss safety issues, particularly the concern regarding thrombotic complications.

**Methods:**

An electronic search was performed in PubMed and the Cochrane Library to identify clinical trials, safety reports and review articles.

**Findings:**

TXA reduces bleeding in patients with menorrhagia, and in patients undergoing caesarian section, myomectomy, hysterectomy, orthopedic surgery, cardiac surgery, orthognathic surgery, rhinoplasty, and prostate surgery. For dental extractions in patients with bleeding disorders or taking antithrombotic drugs, as well as in cases of idiopathic epistaxis, tonsillectomy, liver transplantation and resection, nephrolithotomy, skin cancer surgery, burn wounds and skin grafting, there is moderate evidence that TXA is effective for reducing bleeding. TXA was not effective in reducing bleeding in traumatic brain injury and upper and lower gastrointestinal bleeding. TXA reduces mortality in patients suffering from trauma and postpartum hemorrhage. For many of these indications, there is no consensus about the optimal TXA dose. With certain dosages and with certain indications TXA can cause harm, such as an increased risk of seizures after high TXA doses with brain injury and cardiac surgery, and an increased mortality after delayed administration of TXA for trauma events or postpartum hemorrhage. Whereas most trials did not signal an increased risk for thrombotic events, some trials reported an increased rate of thrombotic complications with the use of TXA for gastro-intestinal bleeding and trauma.

**Conclusions:**

TXA has well-documented beneficial effects in many clinical indications. Identifying these indications and the optimal dose and timing to minimize risk of seizures or thromboembolic events is work in progress.

## Background

**Fig. 1:**
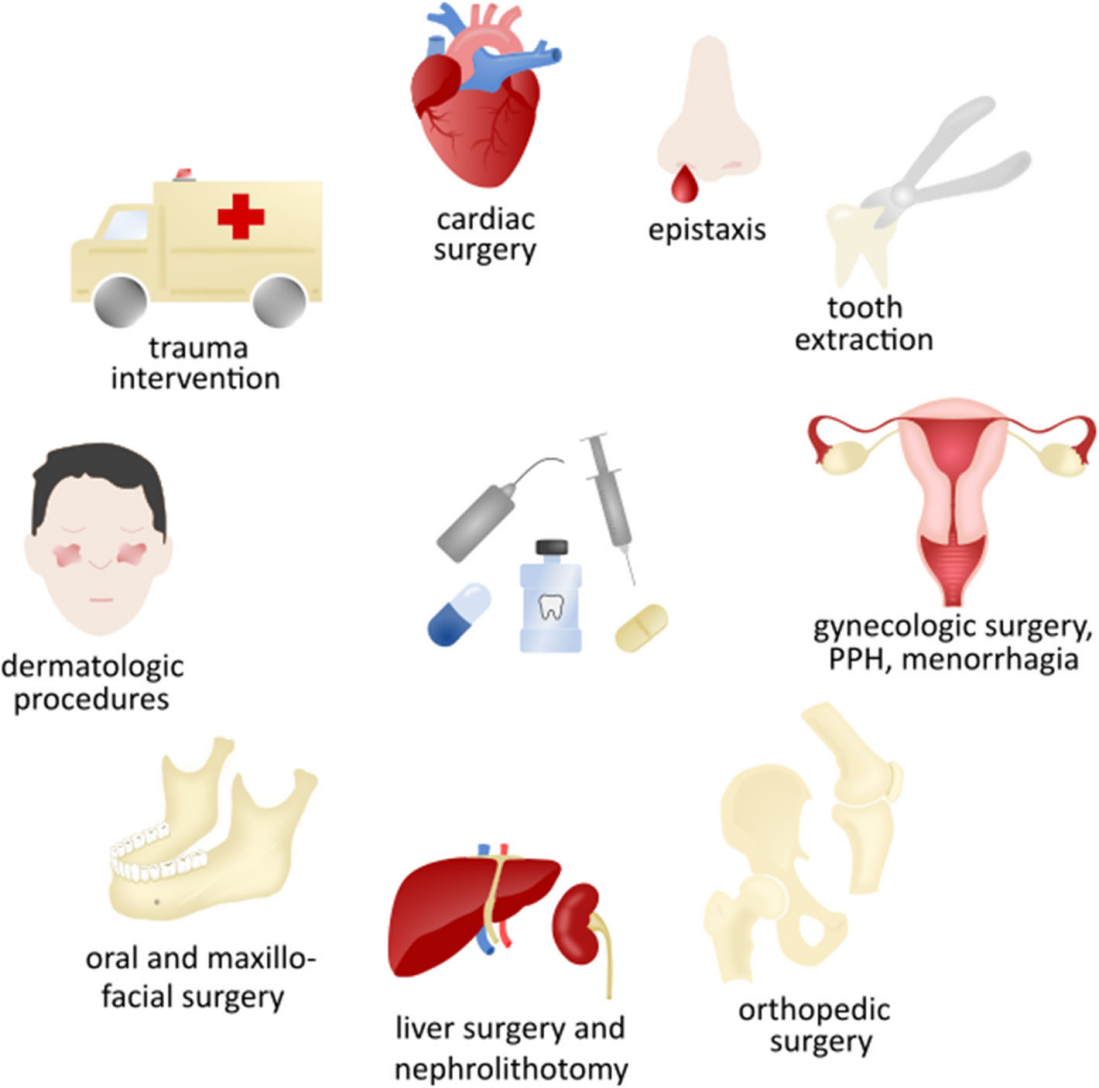
Tranexamic acid can be used to treat or prevent bleeding in various indications. Tranexamic acid is used for prevention and treatment of bleeding in cardiac surgery, epistaxis, gynecologic interventions, postpartum hemorrhage (PPH), menorrhagia, orthopedic surgery, liver surgery and nephrolithotomy, oral and maxillofacial surgery and dental extractions, dermatologic procedures, trauma and bleeding disorders. TXA can be administered orally, intravenously, topically irrigated or on gauzes, and as a mouthwash

Tranexamic acid (TXA) is a fibrinolytic inhibitor that is commonly used in patients with underlying bleeding disorders. Because of hemostatic activity and limited side effects, it has also been widely studied for the prevention and treatment of hemorrhage in trauma and several types of elective surgery and in patients who take antithrombotic drugs undergoing procedures (Fig. 1) [1]. Numerous trials have studied its efficacy in preventing bleeding or reducing bleeding severity, including fatal bleeding. In addition, the drug may reduce the need for transfusion [2]. TXA has limited side effects, but the risk of thromboembolic events with the use of TXA remains uncertain. Because of the importance of TXA in the management of bleeding, the World Health Organization has added TXA to the list of essential medicines [3].

Three antifibrinolytic drugs have been used over the past decades to treat bleeding [4]. ε-Aminocaproic acid (EACA) and TXA are lysine analogues that bind to plasminogen and reduce the binding of plasminogen to fibrin, thereby inhibiting fibrinolysis, whereas aprotinin is a reversible serine protease inhibitor that inactivates free plasmin. TXA is better tolerated and ten-fold more potent than EACA [1]. Aprotinin became unavailable when a clinical trial associated its use with increased overall 30-day mortality in patients undergoing cardiac surgery, but has been reapproved on the market for restricted indications [5, 6]. Because the use of aprotinin is restricted to cardiac surgery and because TXA has a more attractive profile compared with EACA, this review focuses on TXA.

Despite the extensive research on TXA, there are still uncertainties. First, the clinical evidence for TXA in some indications is lacking or ambiguous. Second, the thrombotic risk with TXA remains unclear. Last, there is no consensus regarding the optimal dose in various indications. Therefore, the aim of this narrative review is to provide an overview of the efficacy and safety of TXA in trauma, bleeding disorders and surgery, to indicate where evidence is inadequate and to give an overview of the recommended doses.

### Hemostasis, fibrinolysis and TXA

Hemostasis is established through interactions between platelets, coagulation factors, and the generation of thrombin, and results in a platelet plug that is stabilized via the conversion of fibrinogen to fibrin and the crosslinking of fibrin [7, 8].

Fibrinolysis is catalyzed by the conversion of plasminogen into plasmin, which is mediated by tissue plasminogen activator (tPA) and urokinase plasminogen activator (uPA). Both tPA and uPA stimulate the conversion of plasminogen to plasmin. Active plasmin cuts the cross-linked fibrin strands into fibrin degradation products, resulting in the breakdown of blood clots [9, 10].

TXA is a lysine analogue that impedes the binding of plasminogen to fibrin by blocking (the kringles of) plasminogen. This blockage leads to a powerful inactivation of tPA-driven plasminogen activation. In case of uPA, this mechanism is less efficient, simply because uPA does not bind to fibrin. When the activation of plasminogen to plasmin is inhibited, the process of fibrinolysis is impaired, which results in a more stable blood clot.

### Methods

PubMed and the Cochrane Library were screened until May 2021 via an electronic search query based on the terms tranexamic acid, bleeding, trauma and surgery. Reviews and randomized clinical trials studying the efficacy and safety of TXA in various indications were considered. Wherever possible, we collected the incidence of thrombotic complications from individual studies that reported on thrombotic complications, or from reviews focusing on specific indications. Thrombotic events were defined using study-specific definitions.

## Maintext

### Clinical indications for the use of TXA

 TXA was first used in patients with hereditary bleeding disorders, such as hemophilia and von Willebrand disease, and for hemorrhage in the oral cavity and nose. Thereafter, studies investigating TXA for menorrhagia and postpartum bleeding were carried out. Today, the use of TXA has expanded to trauma, emergency and elective surgery, though these indications are still debated.

TXA can be administered orally, intravenously or topically. Its oral bioavailability ranges from 30 to 50 % and the renal clearance of TXA exceeds 95 %. Its half-life in an adults’ circulation is about 3 h, but its half-life at tissue level can be much longer, up to 17 h [11]. Various dosing schemes of TXA have been proposed, depending on the indication for its use, the route of administration, the patient’s age and weight and the presence of renal insufficiency.

Here, the evidence for the use of TXA per clinical indication is critically reviewed, and a comparison of both TXA in various doses and TXA versus other treatment options is made.

### Bleeding disorders

TXA is recommended in hemophilia patients for the prevention of bleeding caused by dental extraction. One randomized clinical trial (RCT) showed a significant reduction in the number of bleeding events and the need for transfusion in patients treated with TXA compared to patients not treated with TXA [12], and several non-randomized trials supported that TXA minimizes the risk of bleeding [13–15]. Furthermore, it was shown that TXA reduced the need for therapeutic clotting factor concentrates [13–15]. Good hemostasis was achieved by both systemic TXA administration prior to dental extraction and topical TXA application via impregnated gauzes or mouthwash after dental extraction. The recommended prophylactic doses are 1 to 1.5 g orally 2–3 times a day, 10 mg/kg intravenous bolus dose before dental extraction, 10 mg/kg intravenous 3 to 4 times a day (inpatients), or 10 mL 5 % mouthwash for 4 times a day after dental extraction (Table 1) [42]. Even though this one RCT (performed in 1972) with a small sample size [12] may seem insufficient to draw conclusions on the definite efficacy of TXA for dental extractions in hemophiliac patients, there is clinical confirmation for its use in patients with bleeding disorders undergoing dental extraction [12–15]. Hence, the use of TXA in hemophiliac patients to minimize bleeding is recommended in several guidelines and has become common clinical practice [57, 58].

**Table 1:** TXA doses found to be beneficial regarding bleeding outcomes for various indications

Indication	Patient	Recommended TXA dose			References
Epistaxis	Regular	PERI		1% soaked pledgets or sprayed	[16–18]
Menorrhagia	Regular or bleeding disorder	PERI		2-4 g/d (max. 5 d)	[19]
Trauma	Regular	PRE		1 g (over 10 min)	[20]
		PERI		1 g (over 8 h)	
Postpartum hemorrhage	Regular	PRE		1 g (over 10 min)	[21]
		POST		1 g if bleeding continuous after 30 min or if bleeding restarts after 24 h	
Myomectomy and hysterectomy	Regular (A)	PRE		10-15 mg/kg	[22–26]
		PERI (optional)		1 mg/kg/h (continuous)	[23, 24]
	Regular (B)	PERI		2 g soaked gauzes	[24, 26]
Orthopedic	Regular	PRE		10-15 mg/kg (max 1.2 g, over 30 min)	[27, 28]
		PERI (optional)		1-3 g diluted in 30-100 mL NS (topical with closure)	[27–30]
		POST		10-15 mg/kg (after 3 hours or via infusion), optionally repeated	[27, 28, 31–35]
Cardiac surgery	Low bleeding risk	PRE		10-15 mg/kg	[36]
		PERI		1 mg/kg/h continuous infusion (6-8 hours)	
	High bleeding risk	PRE		30 mg/kg	
		PERI		16 mg/kg/h continuous infusion (6-8 hours)	
Maxillofacial surgery	Regular (A)	PRE		10-20 mg/kg	[37–39]
	Regular (B)	PERI		1% irrigation	
Minor oral surgery	Anticoagulant or bleeding disorders	PERI (optional)		5% irrigation	[40, 41]
		POST		10 mL 5% mouthwash (4x/d for 2-7 d)	[40–43]
	Bleeding disorders (A)	POST		15-25 mg/kg or 1-1.5 g 2-3x/d	[42, 43]
	Bleeding disorders (B)	PRE		10 mg/kg	
		POST (optional)		10 mg/kg 3-4x/d	
(Adeno) tonsillectomy	Regular (A)	PRIOR		10 mg/kg	[44, 45]
		POST (optional)		250 mg 3x/d for 1d and 500 mg 1x/d for 2 d	
	Regular (B)	POST		4% irrigation	
Rhinoplasty	Regular (A)	PRE		10 mg/kg	[46]
	Regular (B)	PRE		1 g	
		POST (optional)		1g 3x/d (up to 5 d)	
Liver transplantation and resection	Regular	PERI		10-40 mg/kg/h	[47, 48]
Prostate surgery	Regular (A)	PERI		2 g 3x/d	[49, 50]
		POST		2 g 3x/d (first postoperative day)	
	Regular (B)	PERI		1 g diluted in 200 mL NS or 10 mg/kg for 0.5 h	
	Regular (C)	PRE		500 mg injection	
		PERI		250 mg/h	
	Regular (D)	PRE		500 mg injection	
		POST		500 mg injection	
	Regular (E)	PERI		500 mg irrigation	
Nephrolithotomy	Regular (A)	PRE		1 g	[51, 52]
		POST		500 mg 3x/d for 1 d	
	Regular (B)	PERI		0.1% irrigation	
Melasma	Regular (A)	PERI		40-500 mg/d 2 x/d (for 12 weeks)	[53–55]
	Regular (B)	PERI		10% topical or with microneedling 2 x/d (for 12 weeks)	
Mohs micrographic surgery	Regular	PERI		100-600 mg subcutaneous	[56]
Burn wounds or skin grafting	Regular	PRE		15 mg/kg	

*with d* day(s), *min* minutes, *h* hours, *pre* preoperative dose, *peri* perioperative dose, *post* postoperative dose, *NS* normal saline,  = oral dose,  = intravenous dose,  = topical administration or irrigation,  = mouthwash. (A), (B), (C), (D) and (E) are different dose regimen options of TXA for the same indication

 For patients with von Willebrand disease (VWD), no RCTs studying TXA for dental extractions are available [42], but it is likely that patients with VWD benefit from TXA as well. This is supported by a study by Hewson et al., that include both VWD and hemophiliac patients and reported excellent postoperative hemostasis with local application of TXA [13]. Additionally, guidelines from the *UK Hemophilia Centre Doctors’ Organization* recommend TXA for dental extractions and surgery in VWD patients [43]. The advised doses correspond to the dose recommendations for hemophilia patients (Table 1) [43].

TXA is used with the aim to halt epistaxis in patients with hereditary hemorrhagic telangiectasia. Recurrent epistaxis is a hallmark of this disease and negatively affects the quality of life of the patients. However, a meta-analysis showed that oral or topical TXA modestly but not significantly reduced the frequency and severity of epistaxis compared to placebo, bevacizumab or estriol [59]. TXA is also used for idiopathic epistaxis in inpatients or outpatients visiting the emergency department, especially in patients taking antithrombotic drugs. Moderate evidence has shown that topical TXA significantly reduced the duration of epistaxis and the risk of re-bleeding (within 10 days) compared to nasal packing [16–18]. For the treatment of epistaxis, TXA can be applied via TXA-soaked cotton pledgets or sprayed, followed by external compression for 15 min (Table 1) [16–18]. Treatment with TXA was also associated with an earlier discharge of the emergency department [18].

### Menorrhagia

TXA has been approved for the treatment of heavy menstrual bleeding [60], which is defined as more than 80 mL blood loss per menstrual cycle. A Cochrane Review found evidence for a 40 to 50 % reduction of the amount of blood loss per menstrual cycle with the use of TXA compared to women given placebo or other treatments, including progestogens, non-steroidal anti-inflammatory drugs, herbal remedies, ethamsylate and a levonorgestrel intrauterine device [19]. There were no data reported in the review on the frequency of thrombotic events [19]. Only oral norethisterone acid 5 mg TID for 10 days between day 14 and 23 of the menstrual cycle) showed a significant greater reduction in bleeding compared to oral TXA (4 doses of 1 g/day between day 1 and 4 of the menstrual cycle) [61]. However, a questionnaire pointed out that patients receiving TXA were equally satisfied with their treatment as patients receiving norethisterone acid [61].

The recommended oral TXA dose for menorrhagia is 2 g/day (low dose) or 3–4 g/day (regular dose) starting on the first day of the period (Table 1) [19].

### Obstetrics and gynecologic surgery

The WOMAN (World Maternal Antifibrinolytic) trial was the largest RCT that studied the efficacy and safety of TXA in the prevention and treatment of postpartum hemorrhage. The trial included 20,060 women undergoing vaginal birth or caesarean section, and compared 1 g intravenous TXA loading dose to placebo in women with established postpartum hemorrhage (Table 1) [21]. If bleeding continued after 30 min or if bleeding stopped and restarted in less than 24 h after the administration of the first dose, a second intravenous dose of 1 g TXA or placebo was given. The results showed that TXA did not impact the all-cause mortality. Bleeding-related death was significantly reduced in the TXA group, but only by 0.4 % (155/10,036 (1.5 %) TXA-treated patients vs. 191/9985 (1.9 %) patients in the placebo group died with RR 0.81; 95 % CI [0.65–1.00]; *p* = 0.045), and only when administered within 3 h of giving birth [21, 62, 63]. This 3-hour window of maximal efficacy is in accordance with the use of TXA in trauma patients in the CRASH-2 trial [20]. The need for transfusion and hysterectomies did not differ between TXA and placebo in the WOMAN trial [21].

Another randomized clinical trial randomizing 4,551 women who underwent caesarean section to 1 g intravenous tranexamic acid or placebo showed that TXA reduced post-partum hemorrhage. Post-partum hemorrhage was recorded in 556 of 2086 women (26.7 %) in the tranexamic acid group and in 653 of 2067 (31.6 %) in the placebo group (adjusted risk ratio, 0.84; 95 % CI [0.75–0.94]; *p* = 0.003) [64]. The need for transfusion and hysterectomies did not differ between TXA and placebo [64]. Importantly, the number of adverse events, including thrombotic events was not increased with TXA compared to placebo [21, 64].

Other treatments for postpartum hemorrhage were compared to TXA. It was shown that both intramuscular prostaglandin analogues (0.25 mg every 15–90 min to a maximum of 8 doses) and rectally administered misoprostol (5 doses of 200 µg) were as effective in reducing bleeding as intravenous TXA, and sublingually administered misoprostol (600 µg) was significantly less effective [65–67].

TXA was also proven effective for prompt bleeding cessation in other gynecologic interventions. Four RCTs on patients undergoing myomectomy showed that TXA significantly reduced bleeding and reduced the need for blood transfusion compared to placebo [22–24, 68], whilst one placebo-controlled RCT (with the lowest samples size (n = 60)) showed no difference in bleeding [69]. TXA also significantly reduced bleeding with hysterectomy, but it is not clear whether or not TXA reduced the need for transfusion, as most studies did not report this outcome variable [25, 26]. The studied doses for both indications varied between 10 and 15 mg/kg intravenous bolus injection prior to surgery, possibly followed by a continuous infusion of 1 mg/kg/h during surgery or a topical (irrigation) dose of 2 g in 100 mL normal saline (Table 1). No thrombotic complications were reported in the reviewed studies on myomectomy and hysterectomy [22–26].

### Trauma

Following the proven benefit and good tolerability of TXA in bleeding disorders, the impact of TXA in trauma and a broad range of surgical interventions was studied.

The role of TXA in trauma was examined in multiple studies. The CRASH-2 (Clinical Randomization of an Antifibrinolytic in Significant Hemorrhage 2) trial randomized 20,211 trauma patients to receive either an intravenous TXA loading dose of 1 g over 10 min followed by intravenous TXA 1 g over 8 h or a matching placebo (Table 1) [20]. The results showed an absolute mortality reduction of 1.5 % and a relative risk (RR) reduction of about 9 %; RR 0.91; 95 % CI [0.85–0.97]; *p* = 0.0035. All-cause mortality at 28 days was observed for 1463 of 10,060 (14.5 %) TXA-treated patients and in 1613 of 10,067 (16.0 %) patients in the placebo group. Bleeding-related death was reduced when TXA was given until 3 h after the initial injury, but was increased if TXA was given more than 3 h thereafter. There were 198 of the 3747 (5.3 %) patients in the tranexamic acid group and 286 of the 3704 (7.7 %) patients in placebo group who died due to bleeding if treatment was given within 1 h from injury (RR 0.68, 95 % CI 0.57–0.82; *p* < 0.0001), and, respectively, 147/3037 (4.8 %) and 184/2996 (6.1 %) if treatment was given between 1 and 3 h (RR 0.79, 95 % CI [0.64–0.97]; *p* = 0.03). In the group of patients that received treatment after 3 h, 144/3272 (4.4 %) patients treated with TXA and 103/3362 (3.1 %) patients receiving placebo died due to bleeding (RR 1.44, 95 % CI [1.12–1.84]; *p* = 0.004) [20, 63]. No significant differences in bleeding and number of blood transfusions between TXA and placebo were observed and the risk of thrombotic events was similar [20].

The CRASH-2 trial investigated a population with low injury severity and low rates of penetrating trauma. Other studies indicated that TXA might be harmful in other patient populations. An increased mortality was observed when treating critically injured patients who suffer penetrating trauma and trauma patients with physiologic levels of fibrinolysis with TXA [70, 71]. The TAMPITI randomized controlled trial of 150 severely injured patients found no differences in mortality or transfusions between TXA and placebo, and, moreover, showed an increased incidence of thromboembolic events compared to the placebo group (placebo, 12.0 %; 2 g TXA, 26.5 %; 4 g TXA, 32.0 %; *p* = 0.05) [72]. This latter finding is in agreement with a retrospective study showing that TXA is associated with an increased odd of venous thromboembolism (OR 3.3; 95 % CI [1.3–9.1]; *p* = 0.02) [73].

Concerning patients with brain injury, no trials exist that convincingly demonstrated that TXA reduces brain bleeding. A systematic review showed that TXA after brain injury likely has no effect on mortality or disability [74–76]. This finding was supported by a randomized clinical trial, that included 1,603 patients randomized over two different TXA dosing regimens and placebo, showing that TXA does not improve neurologic outcome at 6 months in patients with moderate to severe traumatic brain injury [77]. On the other hand, results of the CRASH-3 (Clinical Randomization of an Antifibrinolytic in Significant Head injury 3) trial suggest that the use of 1 g TXA intravenous loading plus 1 g TXA maintenance dose in brain injury patients may reduce head injury-related death [78]. However, this reduction was only seen in patients with mild to moderate head injury (RR 0.78, 95 % CI [0.64–0.95]) and not in patients with severe head injury (RR 0.99, 95 % CI [0.91–1.07]) [78]. In the CRASH-3 trial the risk of thrombotic complications (RR 1.08, 95 % CI [0.71–1.64]) and seizures (RR 1.09, 95 % CI [0.90–1.33]) was similar between the TXA and placebo groups [78].

The only outcome for which TXA might be beneficial is growth of the hemorrhagic mass. Multiple studies indicated that TXA, in the same dose as in CRASH-2, reduces intracranial hemorrhage progression compared to placebo-treated patients [75, 76, 79–81]. The clinical relevance of this finding, however, is unclear, given the negative results of the aforementioned studies [74–77]. Further, oral TXA and oral Σ-aminocaproic acid were compared for the treatment of intracerebral hemorrhage, but no difference was found in their effect [82].

Recent studies focused on the use of TXA for trauma patients in a pre-hospital setting. The STAAMP trial evaluated the use of TXA in 927 injured patients [83, 84]. The trial showed that TXA did not lower 30-day mortality in the overall study population, but did find benefit in two subgroups: in patients who received TXA within 1 h of injury (4.6 % vs. 7.6 %; difference, -3.0 %; 95 % CI [-5.7–0.3 %]; *p* < 0.002) and in patients with severe shock (systolic blood pressure ≤ 70 mmHg) [83, 84]. When administering TXA within 1 h, patients also had a lower incidence of multiple organ failure and 6-hour and 24-hour transfusion requirements compared to placebo [84]. There was no difference in thromboembolic rates between the TXA and control groups, but a similar subgroup analysis to look for harm as the one to look for mortality benefit was not performed [83]. Another randomized trial evolved 967 patients with suspected traumatic brain injury and showed that the 28-day mortality was the same for patients treated with a 2 g prehospital bolus of TXA, or a prehospital 1 g bolus followed by a 1 g in-hospital infusion of TXA compared to placebo [85]. However, mortality was reduced in a subgroup of patients with a confirmed intracerebral haemorrhage who received a prehospital 2 g bolus of TXA [85].

### Orthopedic surgery

Several RCTs and RCT-based meta-analyses about patients undergoing total hip or knee replacement, shoulder arthroplasty, hip or humeral fractures or spine surgery demonstrated the efficacy of TXA in reducing hemorrhage and transfusion, without increasing the risk of deep vein thrombosis [86–91]. However, there are studies indicating a concerning increase in inflammation and joint fibrosis resulting from the use of TXA [92, 93].

Concerning the route of administration. Two meta-analyses showed that a combined intravenous and topical TXA administration is superior to an intravenous dose alone for patients undergoing total knee or hip replacement [29, 30]. Additionally, one RCT on total knee replacement showed that the combination of intra-articular and periarticular TXA injections (additional to an intravenous bolus injection) was superior for reducing blood loss and transfusion necessity compared to only giving TXA intra-articular or periarticular [94]. For patients at a higher risk of thrombotic complications, a topical application alone may be preferred [27].

Effective TXA doses range from 10 to 15 mg/kg intravenous bolus dose and optionally 1–3 g (diluted in 30–100 mL normal saline) intra-articularly or topically, followed by another intravenous dose after 3 h or via continuous infusion (Table 1) [27, 28].

Several RCTs compared TXA to other treatment options. First, TXA has been proven to significantly reduce bleeding and transfusion necessity more efficiently than knee flexion in the first hours after surgery, topical application of bovine-derived thrombin combined with fibrinogen solution and electrocoagulation in patients undergoing total knee replacement [95, 96]. Compared to fibrin glue, one study showed significantly less bleeding and transfusion need with TXA [96], whereas two other studies demonstrated no significant difference in blood loss or the need for transfusion [97, 98]. As for the comparison of TXA with Σ-aminocaproic acid, both intravenously administered, one study proved TXA to reduce bleeding more efficiently, while another study showed no significant difference. Both studies demonstrated an equal transfusion need [99, 100]. Second, for patients undergoing spine surgery, one study compared intravenous TXA (bolus of 20 mg/kg and 10 mg/kg/hour maintenance dose) with intravenous batroxobin (0.02 U/kg, repeated every 2 h) and noted no significant difference in bleeding and transfusion between both groups [101]. Last, in patients with a hip fracture, topical applicated TXA (1 g) and fibrin glue (10 ml) resulted in comparable blood loss and transfusion necessity [102].

### Cardiac surgery

TXA is also used during cardiac procedures. The ATACAS (Aspirin and Tranexamic Acid for Coronary Artery Surgery) trial randomized 4,462 patients scheduled for coronary-artery bypass surgery (on-pump or with cardiopulmonary bypass and off-pump) between TXA and placebo [103]. The studied TXA dose was 50–100 mg/kg administered intravenously after the induction of anesthesia and an effective plasma concentration of the drug was maintained for approximately 6 to 8 h. The results demonstrated that TXA reduced blood loss, blood transfusion and reintervention, without increasing the risk of thrombotic complications or death within 30 days after surgery [103].

One large and well-designed RCT suggested that a high dose of TXA (> 80 mg/kg total dose) was more effective in reducing bleeding compared to a low dose (< 50 mg/kg total dose) [104], but two other RCTs did not show any difference between the doses [105, 106]. However, these latter studies excluded patients at high risk of bleeding, and precisely those patients might benefit from higher TXA doses [36]. Therefore, some consider the use of a lower dose (10 mg/kg bolus + 1 mg/kg/h infusion) for patients at low risk of bleeding, and reserve a higher dose (30 mg/kg bolus + 16 mg/kg/h infusion) or multiple low doses for high-risk patients (Table 1) [36]. However, in practice, it can be difficult to adequately assess the bleeding risk of a given patient [36].

Besides the positive results of TXA, some studies suggest there is an association between a high TXA dose (a total dose of > 80 mg/kg) and a risk of postoperative seizures [36, 107–109]. More about this in the discussion part of the paper.

Several RCTs compared TXA to aprotinin and Σ-aminocaproic acid for the reduction of bleeding and transfusion necessity with cardiac surgery. Most studies did not show a significant difference in blood loss and transfusion need between TXA and aprotinin [110–116]. A Cochrane Systematic Review comparing the use of TXA, aprotinin and Σ-aminocaproic acid on the other hand, pointed out an advantage of aprotinin over the TXA and Σ-aminocaproic acid in terms of operative blood loss and the need for a blood transfusion, but stressed that the differences were small [2]. In addition, some data do not exclude an association between aprotinin and increased risk of renal failure [2, 117], and TXA and Σ-aminocaproic acid are cheaper alternatives. Regarding TXA versus Σ-aminocaproic acid, there are contradictory results amongst studies: some indicated the superiority of TXA, some of Σ-aminocaproic acid and others showed no difference in terms of bleeding reduction [116, 118–120]. No difference in transfusion between TXA and Σ-aminocaproic acid was found [116, 118–120].

### Oral and maxillofacial surgery

Various meta-analyses based on RCTs studying TXA in orthognathic surgery showed that intravenous or topical TXA significantly reduced intraoperative bleeding and blood transfusion [37–39]. A more recent RCT (2020) that was not included in these meta-analyses confirmed these results [121]. There were no data reported regarding the number of thrombotic events [37–39], except in the most recent RCT that reported no venous thromboembolic complications [121]. It was also shown that TXA reduces bleeding compared to placebo in patients who underwent mandibular fracture surgery [122]. Both a single preoperative intravenous dose of 10–20 mg/kg TXA or 1 % TXA irrigation appeared effective (Table 1) [37–39, 121].

In addition to orthognathic surgery, TXA can also be applied during minor oral surgery, such as dental extractions, especially in anticoagulated patients. TXA was shown to be effective in reducing bleeding with minor oral surgery in patients on continued vitamin K antagonists and continued antiplatelet therapy without increasing the thrombotic risk [40, 41]. TXA irrigation of the surgical site in combination with the postoperative use of TXA mouthwash appeared to reduce bleeding (Table 1), more than without topical irrigation [40, 41]. The optimal dose regimen of TXA with minor oral surgery in anticoagulated patients is arguable though. A systematic review evaluating hemostatic agents after dental extraction in patients on antithrombotics performed by our group showed that the use of TXA-soaked gauzes was not proven to be better than other hemostatic methods [41]. A two-day and five-day postoperative regimen with 4.8 % TXA mouthwash were proven to be equally effective in patients on continued warfarin [123].

### Adenotonsillectomy and rhinoplasty

Further, TXA can be applied during head and neck surgery, such as (adeno)tonsillectomy and rhinoplasty, as well. For (adeno)tonsillectomy, topical irrigation or intravenous administration of TXA (Table 1) appears to significantly reduce the amount of blood loss, but not the incidence of bleeding [44, 45]. However, there are contradictory results and most studies that investigated (adeno)tonsillectomy were performed before 1980, and future well-designed randomized controlled trials may shed new light on the findings. For rhinoplasty, a meta-analysis including 276 patients indicated that TXA, 10 mg/kg intravenous or 1 g orally prior to surgery, possibly followed by 1 g 3x/day postoperatively for a maximum of 5 days (Table 1), resulted in a significant reduction of intraoperative bleeding (as well as postoperative eyelid edema and ecchymosis) [46]. There are no data available regarding the thromboembolic events [46].

### Gastrointestinal surgery

In liver transplantation, TXA may significantly reduce bleeding and transfusion requirements without increasing the risk of thrombotic events [47, 48]. Studied doses ranged from 2 to 10 and 40 mg/kg/h. It was shown that the 2 mg/kg/h dose did not reduce the need for transfusion, whereas the higher doses did (Table 1) [47, 48, 124].

For patients undergoing liver transplantation, no significant difference in blood loss and transfusion necessity was observed between TXA and aprotinin [125, 126]. For patients undergoing liver resection, TXA reduce blood loss and transfusion more compared with placebo or aprotinin [127, 128].

We should however note that only a small number of trials, with small sample sizes, have studied TXA and/or aprotinin in liver transplantation and resection [128].

Furthermore, a Cochrane review reported on the beneficial effects of TXA on mortality and risk of surgery when used in patients presenting with upper gastrointestinal bleeding, without a difference in the thromboembolic risk between TXA and placebo or control interventions. However, the need for transfusion was not different, and TXA did not significantly reduce re-bleeding in the upper gastrointestinal tract [129]. Of note, a high drop-out rate was reported in some trials. A more recent systematic review (2021) indicated that TXA might decrease the risk of re-bleeding and the need for surgery after upper gastrointestinal bleeding [130].

Until now, no studies showed that TXA decreases blood loss or improves clinical outcomes in patients presenting with lower gastrointestinal bleeding [130, 131]. The thromboembolic risk did not differ between TXA-treated and placebo-treated patients [130, 131]. Only a low number of patients were studied in these trials.

The HALT-IT (tranexamic acid for the treatment of gastrointestinal bleeding) trial with large sample size allows us to draw more powerful conclusions about the use of TXA for upper and lower gastrointestinal bleeding. HALT-IT randomized 12,009 patients to TXA or placebo (1 g loading dose and 3 g maintenance dose over 24 h) or placebo [132]. The trial showed that TXA did not reduce death within 5 days from gastrointestinal bleeding (22 of the 5956 (4 %) patients in the tranexamic acid group and 226 of the 5981 (4 %) patients in the placebo group died, RR 0.99, 95 % CI [0.82–1.18]) and, moreover, was associated with an increased risk of venous thromboembolic events, RR 1.85, 95 % CI [1.15–2·98], and seizures, RR 1.73, 95 % CI [1.03–2.93] [132]. Consequently, the routine use of TXA for this indication is not advised [132].

### Urogenital surgery

In prostate surgery and nephrolithotomy, the use of TXA reduced blood loss and transfusion without increasing the thrombotic risk [49–52]. TXA can be given orally, intravenously or topically for these indications (Table 1). However, the use of TXA with nephrolithotomy is not well studied and there are reports of anuria in patients with a solitary kidney caused by clot obstruction linked to TXA during nephrolithotomy [51, 52]. For this latter reason, caution is advised for the use of TXA.

### Dermatology

Recent studies proved TXA to be useful in patients with melasma [53–55, 133]. By reducing the activation of plasminogen to plasmin, TXA causes a decrease in fibroblast growth factor and prostaglandins, inhibits melanocyte activity and so reduces hyperpigmentation [53]. The studied doses of TXA were 40–500 mg/day orally or 10 % TXA solution 2 times/day for 12 weeks [53–55]. Only one study demonstrated no significant improvement of melasma after topical TXA application compared to placebo, which might be explained by the lower dose of TXA (5 % TXA solution 2 times/day for 12 weeks) used in this RCT [134]. There is uncertainty about which route of administration is best.

Hydroquinone is another treatment option for melasma. One study demonstrated a significant decrease in the severity of melasma in the TXA group [135], while two other studies showed a comparable improvement between TXA and hydroquinone [136, 137]. However, patients treated with TXA had less side effects and better patient satisfaction scores [137].

Moreover, TXA can also be applied for skin procedures. With Mohs micrographic surgery, a procedure used to treat skin cancer, for which it resulted in significantly less blood loss and transfusion necessity compared to placebo [56, 138]. The advised administration in patients undergoing Mohs micrographic surgery was a preoperative subcutaneous administration of 100–600 mg, depending on the tumor size (Table 1) [138]. In patients with burn wounds or undergoing skin grafting, a preoperative intravenous administration of 15 mg/kg was effective (Table 1) [56]. TXA for these indications was however not extensively studied so that final recommendations cannot be made.

### Side effects of TXA

TXA is generally well-tolerated. Nevertheless, some side effects have been reported, such as gastrointestinal complaints (e.g. nausea, diarrhea, vomiting), allergic skin reactions and hypersensitivity reactions [139, 140]. Apart from these rather mild side effects, more severe side effects may occur as well. First, an overly rapid intravenous injection of TXA can lead to hypotension. Second, there might be a risk of TXA-induced thrombotic events. Some cases of thrombotic events following TXA treatment in women (without a cardiovascular risk profile) are reported in the literature, such as aortoiliac thrombosis after TXA usage during hysterectomy [141], pulmonary embolism after TXA for menorrhagia [142], myocardial infarction after TXA for bleeding associated with uterine leiomyoma [143] and deep vein thrombosis after TXA during orthognathic surgery [144]. Additionally, the HALT-IT trial showed that the rate of venous thromboembolism events was twice as high in the TXA group (48/5952 or 0.8 %) compared to the placebo group (26/5977 or 0.4 %, RR 1.85; 95 % CI [1.15–2.98]) [132]. On the other hand, a recent meta-analysis evaluating 216 studies could not identify an increased risk of any thromboembolic complications in patients treated with TXA within multiple medical disciplines [145].

Importantly, as mentioned before, the use of high-dose TXA in cardiac surgery with cardiopulmonary bypass is associated with seizures, probably in a TXA dose-dependent way [109, 146]. Furthermore, TXA during nephrolithotomy in patients with a solitary kidney may cause clot anuria [51, 52]. Finally, TXA can accumulate in the kidneys of patients with (chronic) kidney disease or kidney transplantation and induce toxicity [147].

## Discussion

This review navigates physicians through the clinical evidence for the use of TXA in bleeding disorders, trauma, and various surgical interventions. The efficacy and tolerability of TXA in bleeding disorders prompted its evaluation in other indications. We highlighted where evidence is lacking, compared TXA to other treatment options, and we looked into the various TXA dosages and associated complications.

The use of TXA resulted in a reduced bleeding-related mortality when applied in postpartum hemorrhage and trauma patients, as shown in the WOMAN and CRASH-2 trials, without evidence for an increased risk of thrombotic complications [20, 21]. The absolute reductions in mortality, however, were moderate (0.4 and 1.5 % respectively) and a delayed treatment reduced the benefit of tranexamic acid administration [62, 63]. Hence, patients should be treated with the shortest delay possible, within 3 h after the onset of bleeding. Importantly, both the WOMAN and CRASH-2 trials were mostly performed in low- and moderate-income countries, which raises concerns about the applicability of the results to patients treated in more-developed trauma centers [20, 21]. Per contra, the authors of the CRASH-2 trial demonstrated no significant heterogeneity in terms of bleeding-related mortality between different geographic regions (Africa, Asia, Europe, North America, Australia and South America) [20].

As for TXA’s effectivity for other indication than trauma and postpartum hemorrhage. TXA improves bleeding outcomes in cases of menorrhagia, myomectomy, hysterectomy, orthopedic surgery, cardiac surgery, orthognathic surgery, rhinoplasty, prostate surgery and melasma. Moreover, it was shown that TXA reduced transfusion rates in cases of myomectomy, orthopedic surgery, cardiac surgery, orthognathic surgery, liver transplantation and resection, prostate surgery and nephrolithotomy. For dental extractions in patients with bleeding disorders or taking antithrombotic drugs, as well as in cases of idiopathic epistaxis, tonsillectomy, liver transplantation and resection, nephrolithotomy, Mohs micrographic surgery, burn wounds and skin grafting, there is moderate evidence that TXA is effective for reducing bleeding.

Elseways, at present, there is insufficient evidence to support the efficacy of TXA for reducing bleeding in epistaxis in patients with hereditary hemorrhagic telangiectasia, although this is common clinical practice. Further, no consistent significant effect of TXA was found in patients with traumatic brain injury and upper and lower gastrointestinal bleeding.

Further, the data to support the use of TXA in dental extractions in patients with hemophilia or VWD and in tonsillectomy is somewhat outdated, so high-quality studies might be appropriate to support these findings. In addition, TXA mouthwash reduced bleeding after dental extraction in patients on vitamin K antagonists and antiplatelet drugs, but there are no studies supporting its use in patients on other types of antithrombotic drugs, except for one. The recent randomized, placebo-controlled, randomized EXTRACT-NOAC trial, indicated that the use of TXA may reduce the risk of delayed bleeding and bleeding with multiple dental extractions in patients treated with non-vitamin K oral anticoagulants [148].

It remains unclear if TXA is more effective for specific causes of bleeding or whether selection of patients, dose, route of administration or other methodological reasons explain the different outcomes for various indications. Even in the absence of strong clinical evidence, several guidelines recommend the use of TXA in life-threatening bleeding regardless of the origin, based on the convincing results in some indications and the low risk of adverse events.

As for the complications with the use of TXA, there is a concern for thrombotic complications. On the one hand, most randomized trials do not signal an increased risk for thrombotic events, but on the other hand, some trials reported an increased rate of thrombotic complications with the use of TXA for gastro-intestinal bleeding and trauma and several anecdotal case reports of thrombotic complications after TXA use can be found in the literature. Hence, its administration in patients who have recently presented with acute thrombotic events should be carefully evaluated. Importantly, bleeding itself may induce a hypercoagulable state and increase the risk of thrombosis.

Another TXA-associated complication is the dose-dependent risk of seizures after TXA administration during cardiac surgery with cardiopulmonary bypass. The seizures might be explained by the inhibitory effect of TXA on gamma-aminobutyric acid type A (GABA-A) and glycine receptors, which are two major mediators of central nervous system inhibition. Inhibiting these receptors might cause synaptic excitation and increase the risk of seizures [149]. Studies on the dose of TXA and associated side effects in cardiac surgery should allow us to find out more about this risk.

Last, there are reports of clot anuria associated with TXA when administered for reducing bleeding during nephrolithotomy in solitary kidney patients. In any case, caution is advised in patients suffering from renal insufficiency, as standard dosing of TXA in these patients might lead to a higher blood concentration of TXA when compared to patients with normal renal function. Additionally, TXA might accumulate in the kidneys of these patients and induce nephrotoxicity [147]. For this reason, dose adjustments are required for patients with renal insufficiency (Table 2).

**Table 2:** TXA dose adaptations for patients with renal insufficiency for various indications

Indication for TXA	Patient	Recommended TXA dose			References
Menorrhagia	Minor RI	PERI		1.3 g 2x/d (max. 5 d)	[150, 151]
	Mild RI	PERI		1.3 g/d (max. 5 d)	
	Severe RI	PERI		0.65 g/d (max. 5 d)	
Orthopedic	Minor RI	PRE		10-15 mg/kg (max 1.2 g, over 30 min)	[151]
		PERI		11.25 mg/kg (8h and 16h after first dose)	
	Mild RI	PRE		10-15 mg/kg (max 1.2 g, over 30 min)	
		PERI		8.4 mg/kg (8h and 16h after first dose)	
	Severe RI	PRE		10-15 mg/kg (max 1.2 g, over 30 min)	
		PERI		6.3 mg/kg (8h and 16h after first dose)	
Cardiac surgery	Low bleeding risk + minor RI	PRE		10-15 mg/kg	[151, 152]
		PERI		3.75 mg/kg/h continuous infusion	
	Low bleeding risk + mild RI	PRE		10-15 mg/kg	
		PERI		2.50 mg/kg/h continuous infusion	
	Low bleeding risk + severe RI	PRE		10-15 mg/kg	
		PERI		1.25 mg/kg/h continuous infusion	
	High bleeding risk + minor RI	PRE		25-30 mg/kg	
		PERI		11-16 mg/kg/h continuous infusion	
	High bleeding risk + mild RI	PRE		25-30 mg/kg	
		PERI		5-10 mg/kg/h continuous infusion	
	High bleeding risk + severe RI	PRE		25-30 mg/kg	
		PERI		3-5 mg/kg/h continuous infusion	
Minor oral surgery	Minor RI	POST		10 mg/kg 2x/d	[150, 151]
	Mild RI	POST		10 mg/kg 1x/d	
	Severe RI	POST		10 mg/kg 1x/2d or 5 mg/kg 1x/d	

*with d* day(s), *min* minutes, *h* hours, *pre* preoperative dose, *peri* perioperative dose, *post* postoperative dose, *NS* normal saline, *RI* renal insufficiency with minor RI when GFR = 60-89 mL/min/1.73m^2^; mild RI when GFR = 30-59 mL/min/1.73m^2^; severe RI when GFR < 29 mL/min/1.73m^2^ and GFR = glomerular filtration ratio.

= oral dose,  = intravenous dose

The advised TXA dose regimen is quite straightforward in some cases, such as in trauma or postpartum hemorrhage, in contrast to some other indications where TXA can be applied following various regimens and routes of administration. In this paper, we list numerous doses that were found effective in reducing or treating bleeding (Table 1). However, TXA is actually only FDA (Federal Drug Agency)-approved for treating menorrhagia and preventing bleeding in patients with hemophilia. The EMA (European Medicines Agency), on the other hand, approved the use of TXA for a broader range of indications, including menorrhagia, gastrointestinal bleeding, prostate, urinary, ear, nose, and throat, abdominal, thoracic, gynecologic and cardiac surgery [153].

Different TXA treatment options are advantageous for patients with a high-risk profile for thrombotic events, for whom TXA can be administered topically instead of intravenously. Indeed, the topical formulation of TXA leads to minimal systemic absorption and consequently a lower risk of systemic side effects than with intravenous TXA. Two examples of topical TXA administration are TXA irrigation of the surgical site during orthopedic surgery and the use of TXA mouthwash after minor oral interventions.

Other advantages of TXA are its broad availability on the market and low cost [154–156].

## Conclusions

In conclusion, TXA is an easy-to-use and high-potential drug with beneficial effects on bleeding and blood transfusion requirements within many fields of medicine. For some indications, evidence for TXA use is (still) lacking, and/or the optimal dose regimen is unclear. Ongoing vigilance for the risk of thrombotic events and possible side effects with the use of TXA is needed.

## Data Availability

Not applicable.

## Abbreviations

CI: Confidence interval
EACA: Aminocaproic acid
EMA: European Medicine Agency
FDA: Federal Drug Agency
RCT: Randomized clinical trial
RR: Relative risk
TID: three times a day
tPA: Tissue plasminogen activator
TXA: Tranexamic acid
uPA: Urokinase plasminogen activator
VWD: Von Willebrand Disease
